# Neurophysiologic tests screening cognitive impairment in idiopathic intracranial hypertension patients

**DOI:** 10.1186/s41983-018-0010-6

**Published:** 2018-04-25

**Authors:** Iman A. Elbanhawy, Gihan M. Ramzy, Mye A. Basheer, Diana M. Khedr

**Affiliations:** 1 Neurology Department, Faculty of Medicine, Cairo University, Manyal, P.C., Cairo, 11553 Egypt; 2 Clinical Neurophysiology Department, Faculty of Medicine, Cairo University, Manyal, P.C., Cairo, 11553 Egypt

**Keywords:** Cognitive impairment, Event-related potentials, Idiopathic intracranial hypertension, Mini-mental state examination

## Abstract

**Background:**

Idiopathic intracranial hypertension (IIH) is a disorder with increased intracranial pressure of obscure cause. Patients with IIH may suffer from difficulty in thinking or concentrating. This work aimed at highlighting the neurophysiologic suggestions of cognitive impairment in IIH patients.

**Methods:**

Twenty patients with IIH—and a similar number of matched control subjects—were examined in this case–control study. The P300 and contingent negative variation (CNV) were performed. Results from both groups were compared.

**Results:**

There were significant lower means of P300 amplitude and CNV amplitude (early and late response) in patients than in controls. Also, there were significant delayed latencies of P300 and CNV in patients than in normal control subjects. Finally, P300 latency was correlated to mini-mental state examination.

**Conclusions:**

We concluded that cognitive affection in IIH is well appreciated at neurophysiologic levels and is related to clinical inputs. We are providing a suggestion of the significant relation between clinical screening (i.e., mini-mental state examination) and NP screening (i.e., P300) of cognitive functions.

**Electronic supplementary material:**

The online version of this article (10.1186/s41983-018-0010-6) contains supplementary material, which is available to authorized users.

## Background

Idiopathic intracranial hypertension (IIH) is a disorder with increased intracranial pressure with no clinical, laboratory, or radiological evidence of intracranial pathology on conventional imaging (Wall 2010). Yet, definite altered cerebrospinal fluid (CSF) dynamics is present (Sinclair et al. 2010). Symptoms include headache, diplopia, pulsatile tinnitus, visual blurring, nausea, and vomiting. Modified Dandy criteria can be used to establish a diagnosis of IIH. Moreover, a cutoff for elevated CSF opening pressure needs to be specified (Friedman and Jacobson 2002). The event-related potential (ERP), P300, is involved in cognitive processing (Thakur et al. 2011), whereas the contingent negative variation (CNV) is another ERP that is speculated to show awareness, attention, decision-making, planning, and conation, as well as readiness for a motor response (Uysal et al. 2014).

### Aim of work

The hypothesis of impaired cognition in IIH patients was adopted. Neurophysiologic (NP) tests were used as tools to screen for the presence/absence of impaired cognition in such patients.

## Methods

Twenty patients (group I) with IIH were examined. Patients were diagnosed according to Dandy criteria for diagnosis of IIH disease. Twenty healthy subjects, matched for age, sex, and level of education, represented the control group (group II). All patients of group I and subjects of group II gave informed consent to share in this study. The study was officially approved by two appropriate ethical committees (intradepartmental and faculty ethical committees).

Group I included patients with a score greater than or equal to 24 on mini-mental state examination (MMSE), having intracranial pressure greater than 25 cmH2O measured in lateral decubitus position, showing normal CSF composition, and the absence of hydrocephalus or mass in imaging. Ages ranged from 18 to 45 years, and education started at 6 years or more. The study excluded patients with major language disturbance; severe physical, auditory, or visual impairment affecting their ability to complete testing; those with a history of alcohol intake or any substance abuse, with evidence of any concomitant medical or metabolic illness known to affect cognition—e.g., thyroid or parathyroid disease, hepatic or renal failure; those with current or prior history of major psychiatric disorder and/or current use of anxiolytic, neuroleptic, or sedative medication; and—finally—those with known comorbidities such as hypertension or diabetes mellitus.

The MMSE assesses subjects’ orientation to time and place, instantaneous memory, short-term memory, serial subtractions or reverse spelling constructional capacities (copying a design), and use of language (Folstein et al. 1975). Clinical assessment was carried out for the patients, and it included general examination, with different systems assessment to exclude any associated medical illness, as well as thorough history taking and full neurological examination, according to the IIH sheet of the local Neurology Department (Additional file 1: Appendix 1).

Neurophysiologic tests performed for both groups included P300, as well as CNV (Additional file 3).

The machine used to record both responses was Neuropack MEB-9200 G/K EP/EMG measuring system four-channel, version 08 (Neuropack M1); manufacturer: Nihon Kohden, Tokyo, Japan). The active electrode was placed at CZ according to the 10–20 international system of electroencephalography electrode placement, the reference electrode was placed over either mastoid process, and the ground electrode was placed on the forehead. For the P300 test, the auditory oddball paradigm was carried out, after determining the hearing threshold for each subject. Two hundred auditory stimuli (bursts) were presented to the ears through earphones, with an intensity of 60 dB above the hearing threshold; 80% of tones were 2 kHz in frequency (background tones), whereas the remaining 20% were 8 kHz (target tones). These tones presented randomly intermixed at a rate of 0.5/s. The wave P300 was defined as the most positive point of the average waveform to the target tones after 250 ms and before 500 ms. However, for the CNV, a single click elicits a brief positive peak and a brief negative peak. On the other hand, when a single click is followed by repetitive flashes that are terminated by a button press, there is another large gradual negative peak, which ends sharply with the button press. This is the CNV methodology used in the study. The CNV appears after about 30 trials of paired stimuli, although this number can be reduced when the subject understands the task in advance.

Statistically, the data were analyzed using IBM SPSS advanced statistics (version 22; SPSS Inc., Chicago, IL, USA). Numerical data were expressed as mean and SD or median and range as appropriate. Qualitative data were expressed as frequency and percentage. *χ*^2^ test or Fisher’s exact test was used to examine the relation between qualitative variables. For non-normally distributed quantitative data, comparison between two groups was done using Mann–Whitney test (nonparametric *t* test). Spearman’s method was used to test correlation between numerical variables. All tests were two-tailed. The descriptive analysis of the results is as follows: The data were summarized using minimum, maximum, mean, and SD for quantitative data and the frequency distribution for qualitative data. The probability/significance value is as follows: *P* value greater than or equal to 0.05 was not significant, whereas *P* value less than 0.05 was significant. A correlation is a single number that describes the degree of relationship between two variables.

The most common type is the Pearson correlation. The sign of correlation coefficient (+, −) defines the direction of the relationship, either positive or negative. A positive correlation coefficient means that as the value of one variable increases, the value of the other variable increases; as one decreases, the other decreases. A negative correlation coefficient indicates that as one variable increases, the other decreases, and vice versa.

## Results

In this study, a retrospective neurophysiologic—noninterventional—case–control study, the age of patients ranged from 19 to 44 years, with a mean value of 32.6 ± 7.7 years, and the age of control subjects ranged from 18 to 44 years, with a mean value of 30.6 ± 7.9 years, showing no statistically significant difference between both groups. With regard to gender, 10% (*n* = 2) of the included IIH patients were male and 90% (*n* = 18) were female. As for control subjects, 20% (*n* = 4) were male and 80% (*n* = 16) were female, showing no statistically significant difference between both groups. The years of education for individuals from both groups showed no statistically significant difference.

All patients were suffering from headache, which ranged in duration from 4 to 50 months, with a mean of 16.6 ± 4.2 months. Eleven (55%) patients had severe headache, with only nine (45%) patients having mild to moderate headache (classification of severity of headache was according to headache pain scale interpretation, Additional file 2: Appendix 2). As regards therapy lines, pharmacologically, monotherapy was used by six (30%) patients receiving steroids in the form of prednisolone 20 mg for a mean of 8 ± 4.5 months and by 13 (65%) patients receiving carbonic anhydrase inhibitor (acetazolamide) for a mean of 30 ± 8 months. Nonpharmacologically, six (30%) patients had lumbo-peritoneal shunt and three (15%) patients underwent optic nerve fenestration. The lipid profile for the patient group is displayed in Table 1.

**Table 1:** Lipid profile of group I

	Minimum	Maximum	Mean ± SD
LDL (mg/dL)	80	146	100 ± 6.6
Cholesterol (mg/dL)	144	250	153 ± 7.4
HDL (mg/dL)	30	77	66 ± 3.8

We compared the results of NP tests in both groups. As regards P300, patients of group I had statistically significant lower amplitude and delayed latency as compared with group II. In addition, as regards CNV, patients of group I had statistically significant lower amplitude and delayed latency of both early and late responses when compared with group II (Table 2).

**Table 2:** Comparison between CNV results in both groups

	Group I mean ± SD	Group II mean ± SD	*P* value
P300			
Amplitude/mv	8.2 ± 4.8	16.7 ± 4.3	0.0239*
Latency/ms	378.8 ± 12.4	320 ± 13.8	0.0139*
Early CNV			
Amplitude (CZ)/mv	6.4 ± 4.3	15.9 ± 4	< 0.001*
Latency/ms	252.7 ± 103.5	130.4 ± 55.2	0.043*
Late CNV			
Amplitude (CZ)/mv	7 ± 5	17 ± 3.9	0.012*
Latency/ms	2248.6 ± 337.9	1244.8 ± 283.3	< 0.001*

*P* value ≥ 0.05 (nonsignificant)

*P value < 0.05 (significant)

Patients who had severe headache had statistically significant lower P300 amplitude and more delayed latency of late CNV response than patients who had mild to moderate headache (Table 3).

**Table 3:** Comparison between results of NP tests in patients of group I as regards severity of headache

Severity of headache			
	Mean ± SD		*P* value
	Mild to moderate (No. = 14)	Severe (No. = 6)	
P300			
Amplitude/mv	12.2 ± 6.3	6.9 ± 3.6	*0.044
Latency/ms	377.8 ± 14.5	378.2 ± 12	0.964
Late CNV			
Amplitude(CZ)/mv	7.4 ± 5.4	6.8 ± 4.2	0.964
Latency/ms	2022 ± 347.7	2357.9 ± 60.9	*0.007

*P* value ≥ 0.05 (nonsignificant)

*P value < 0.05 (significant)

There were no further significant relations. There was statistically significant higher amplitude of the late CNV response for those who were using carbonic anhydrase inhibitor compared with those who were not using it, and there was statistically significant higher amplitude of the early CNV response and earlier P300 latency for those who underwent shunt operation compared with those who did not undergo it. There was no significant difference in all NP tests for those who were using steroids compared with those who were not using it (Table 4).

**Table 4:** Comparison between results of NP Tests in patients of group I as regards different lines of treatment

CAI				Shunt surgery		
	No No. = 7	Yes No. = 13	*P* value	No No. = 14	Yes No. = 6	*P* value
	Mean ± SD			Mean ± SD		
P300						
Amplitude/mv	7.7 ± 4.3	8.4 ± 5.2	0.817	6.5 ± 2.2	8.9 ± 5.5	0.659
Latency/ms	380.4 ± 16.4	377.9 ± 10.2	0.438	387.7 ± 9	375 ± 11.8	*0.026
Early CNV						
Amplitude (CZ)/mv	4.5 ± 2.9	7.4 ± 4.6	0.115	3.2 ± 1.7	7.8 ± 4.4	*0.033
Latency/ms	261.5 ± 96.7	236.3 ± 121.4	0.877	276.7 ± 57.1	242.4 ± 118.4	0.968
Late CNV						
Amplitude (C)/mv	4 ± 2.4	8.6 ± 5.4	*0.046	5 ± 3.9	7.8 ± 5.3	0.207
Latency/ms	2297.3 ± 374.3	2222.3 ± 329.6	0.438	2317.2 ± 364	2088.3 ± 210.1	0.109

*P* value ≥ 0.05 (nonsignificant)

*P value < 0.05 (significant)

When correlating lipid profile to results of NP tests in patients of group I, there was a statistically significant negative correlation between high-density lipoprotein level and P300 latency (*P* = 0.007). There was no significant correlation between lipid profile and other parameters. There was a statistically significant positive correlation between CSF opening pressure and late CNV latency test (*P* = 0.012). There were no significant correlations with other parameters. Finally, there was statistically significant negative correlation between the result of MMSE and P300 latency in group I (*P* = 0.039).

## Discussion

Patients with IIH may suffer from difficulty in thinking or concentrating.

Cognitive function is not addressed routinely during clinical evaluation of IIH patients. An old case report revealed significant cognitive deficits (namely verbal tests and memory) in patients with IIH (Kaplan et al. 1997), whereas a recent study found a more widespread, multi-domain—cognitive—impairment in such patients (Yri et al. 2014). We targeted the NP tests to screen for cognition impairment in IIH patents. All the NP measures of our studies were affected in IIH patients. This was highly supportive to the theory of cognitive affection in such patients, at least at NP levels. Therefore, the P300 arises from multiple areas including the auditory cortex, hippocampus, and amygdala, in addition to brainstem and the thalamic elements (Thakur et al. 2011). In addition, CNV is an ERP that has been related to the activity of the prefrontal cortex and tells about the psychological preparation activities of the subject. It is well correlated with psychological activities such as anticipation, attention, arousal, memory, motivation, preparation, and decision (Uysal et al. 2014). Thus, the findings of our NP study are somewhat aligned with the clinical study, highlighting the association of rather global cognitive dysfunction with IIH (Yri et al. 2014).

Our findings highlighted that the severity of headache, level of serum high-density lipoprotein, use of acetazolamide, and increased CSF opening pressure are the strategic variables in IIH patients affecting cognition at the NP levels. Accordingly, and when considering cognitive impairment in IIH patients, these clinical variables need to be regarded with care and controlled as much as possible through the disease course. The electric events that explain P300 generation originate from the collaboration between frontal lobe and hippocampal/temporal–parietal function. The ERP and fMRI studies using oddball tasks have elaborated patterns supportive to such collaboration. Additionally, magnetic resonance imaging (MRI) suggests that different P300 wave component variations are correlated with frontal lobe area size and parietal area size (Huang et al. 2015). The MMSE, originally conceived for a geriatric population, has been later “borrowed” to—nonspecifically—screen for impaired cognition in patients having different diseases (Corrias et al. 2014).

This implies the nonspecific nature of both tests. The significant correlation between both tests raises the question about possible similar population of brain cells that are called into action during their execution—that is generators. Thus, both tests can interchangeably be used for screening of cognitive impairment, depending on the inputs of the working research plot.

## Conclusions

In conclusion, our study is suggesting that the MMSE, used for basic assessment of cognitive functions, is of strong NP basis. The study also highlighted that cognitive affection in IIH is appreciated at NP levels before being elicited by routine clinical assessment. Neurophysiological assessment of cognitive functioning can assess different aspects of cognitive domains and should be done to detect subtle cognitive decline in IIH patients (Additional file 3). Thus, we are suggesting protective clinical managerial protocols to guard against cognitive impairment of patients diagnosed with IIH, even before clinical evidence of cognitive affection.

## Additional files

Additional file 1: **Appendix 1.** Neurology sheet. (DOC 46 kb)

Additional file 2: **Appendix 2.** Headache Pain Scale Interpretation. (PDF 177 kb)

Additional file 3: Spread sheet of neurophysiologic tests. (XLSX 11 kb)

## Abbreviations

CNV: Contingent negative variation
CSF: Cerebrospinal fluid
ERP: Event-related potential
HDL: High-density lipoprotein
IIH: Idiopathic intracranial hypertension
LDL: Low-density lipoprotein
MMSE: Mini-mental state examination
NP: Neurophysiologic
