# Supplementary material for: Neurophysiologic tests screening cognitive impairment in idiopathic intracranial hypertension patients
Source: Egypt J Neurol Psychiatr Neurosurg. 2018 Apr 25;54(1):7. doi: 10.1186/s41983-018-0010-6 (PMC5954773; doi:10.1186/s41983-018-0010-6)
Supplement: Supplementary file 1 — Appendix 1. Neurology sheet. (DOC 46 kb) [file 41983_2018_10_MOESM1_ESM.doc]

**Appendix (I)**

**Neurology sheet**

- **Personal history:**

-Name:

-Age:

-Sex:

-Marital status:

-Occupation:

-Residence:

-Special habits:

-Handedness:

-Level of education:

- **Menstrual history:**

-Menarche:

-Regularity:

- **Contraceptive history:**

-Hormonal:

IUD:-

- **Obstetric history:**

Number of deliveries:-

Type:-

-Post-operative comp:

- **Past history:**

Diabetes or HTN:-

-Chronic medical illness:

Regular drug intake:-

-Previous operations:

-Previous blood transfusion:

- **Complaint:**
- **Present history:**

**-Headache:**

.onset: .course:

.Duration: .character:

.site: . Referral:

.diurnal variation:

.relation to posture:

.severity:

.association:

.aggravating factors:

.relieving factors:

.ADL:

.analgesic use:

**-visual symptoms:**

.diminution of vision:

.TVOS:

**-Tinnitus:**

**-Any neurological deficit:**

- **General examination:**

-Weight:

-Length:

-BMI:

-Skin:

-Joint:

-Thyroid:

- **Neurological examination:**

-Fundus examination:

-Vision:

-Field examination:

-other cranial nerves:

-other system examination:

- **Investigations:**

-Imaging:

-Labs:

Lipid profile:

CBC:

Coagulation profile:

-lumbar puncture: opening pressure

-perimetry:

- **Treatment:**

Drugs:-

-Shunt:

Lumbar puncture:**-**
