# Supplementary material for: Neurophysiologic tests screening cognitive impairment in idiopathic intracranial hypertension patients
Source: Egypt J Neurol Psychiatr Neurosurg. 2018 Apr 25;54(1):7. doi: 10.1186/s41983-018-0010-6 (PMC5954773; doi:10.1186/s41983-018-0010-6)
Supplement: Supplementary file 2 — Appendix 2. Headache Pain Scale Interpretation. (PDF 177 kb) [file 41983_2018_10_MOESM2_ESM.pdf]

Headache Pain Scale Interpretation

***Please use this information as a reference when completing your headache diaries.**

**10**	Extreme pain; unable to function; worst pain imaginable; bed rest likely required
**9**	Very severe pain; may be unable to speak or think clearly; not able to function; likely lying down or sleeping
**8**	More severe pain; prohibits your activities (i.e., work, child care, self-care, etc.); likely lying down or sleeping
**7**	Severe pain; pain begins to affect ability to concentrate and very difficult to continue with daily activities
**6**	Moderate-severe pain; limits your activities; some activities may be less of a priority
**5**	Moderate pain; you feel the need to slow down; pain is more distracting
**4**	Mild-moderate pain; usual activities become more difficult
**3**	Distracting pain; can continue with usual activities, but may be more difficult
**2**	Mild pain; can continue with daily activities, but may be more difficult
**1**	Very mild pain; able to carry on with usual activities with no distraction
0	Pain free; **no** pain at all; **no headache**
